# Bioactive Collagen Peptides in Veterinary and Biomedical Science—Part II: The Gut–Collagen Peptide Axis, Bile Acid Signaling, and Translational Therapeutic Applications

**DOI:** 10.3390/vetsci13080747

**Published:** 2026-07-28

**Authors:** Krisztián Németh, Borbála Mózes, Tibor Bartha, Boglárka Mária Schilling-Tóth, Gergely Jócsák, Dávid Sándor Kiss, Ágnes Sterczer, Marianna Kis, István Tóth

**Affiliations:** 1 Department of Physiology and Biochemistry, University of Veterinary Medicine Budapest, István u. 2, H-1078 Budapest, Hungary; nemeth.krisztian@univet.hu (K.N.); schilling-toth.boglarka.maria@univet.hu (B.M.S.-T.); jocsak.gergely@univet.hu (G.J.); kiss.david@univet.hu (D.S.K.); toth.istvan@univet.hu (I.T.); 2 Department of Internal Medicine, University of Veterinary Medicine Budapest, István u. 2, H-1078 Budapest, Hungary; mozes.borbala@univet.hu (B.M.); sterczer.agnes@univet.hu (Á.S.); 3 National Laboratory of Infectious Animal Diseases, Antimicrobial Resistance, Veterinary Public Health and Food Chain Safety, University of Veterinary Medicine Budapest, István u. 2, H-1078 Budapest, Hungary; 4 Csepel Central Veterinary Clinic, Táncsics Mihály u. 33, H-1212 Budapest, Hungary; hadakutjan@gmail.com

**Keywords:** collagen hydrolysate, bioactive collagen peptides, gut–collagen peptide axis, bile acid signaling, FXR/TGR5, GLP-1, canine osteoarthritis, taurine-responsive cardiomyopathy, Pro-Hyp, veterinary nutrition

## Abstract

Bioactive collagen peptides and collagen hydrolysates are dietary proteins whose degradation products also carry signaling activity. This second part of a two-part narrative review addresses the translational dimensions of their bioactivity, integrating veterinary clinical trials, controlled animal-model studies, and human biomedical data, with evidence strictly stratified by type. The gastrointestinal tract acts not only as the absorption site but as a target organ. In cell culture and rodent models, luminal collagen fragments restore tight-junction integrity, alter the microbiome, and shift the enterohepatic bile acid pool; increased secondary bile acid synthesis is proposed to engage the farnesoid X receptor (FXR) and Takeda G-protein-coupled receptor 5 (TGR5). None of these steps have been demonstrated in veterinary clinical patients. The accompanying GLP-1 and PYY response may reflect direct amino acid stimulation of enteroendocrine cells as much as a bile acid-dependent route, and current data do not separate the two. Veterinary trials show objective kinetic improvement in osteoarthritic dogs and horses. Taurine-responsive dilated cardiomyopathy is a reversible, diet-amenable condition; as the principal bile acid conjugation substrate in carnivores, taurine acts on the same bile acid pool as a mechanistically separate input. Recent data implicate prolyl-hydroxyproline in brown adipogenesis, collagen peptides in hippocampal neurogenesis, and taurine in platelet normalisation. The gut–collagen peptide axis is an evolving model linking dietary collagen to systemic metabolic, endocrine, and immune signaling, with implications for companion-animal, equine, and livestock practice.

## Simple Summary

Collagen supplements are usually thought of as joint or skin remedies, but the small collagen fragments released during digestion reach far more of the body than the joints alone. This review, the second of two parts, follows these fragments from the gut outward. In the intestine they help seal the gut lining, shift the balance of gut bacteria, and alter bile, the digestive fluid made by the liver. Through these routes they can influence appetite, blood sugar, fat tissue, the liver, and even the brain. We sort the evidence carefully, separating proven clinical results from promising early findings. The firmest veterinary evidence supports collagen fragments for joint disease in dogs and horses, and dietary taurine—a different nutrient that also works through bile—for a reversible form of heart-muscle disease in certain dog breeds. Newer laboratory work points to effects on fat-burning, memory, and blood clotting, but these still need confirmation in animals before any clinical claim. The practical message is that diet shapes a shared gut–bile signalling system, and that collagen and taurine act on it as separate dietary inputs, with real relevance for companion animals, horses, and livestock.

## 1. Introduction

The first part of this two-part review established the molecular identity, gastrointestinal bioavailability, and receptor-mediated signaling mechanisms of low-molecular-weight bioactive collagen peptides (BCPs) [1]. Two of those findings are well supported. Orally administered collagen hydrolysates (CH) resist complete proteolysis and enter the systemic circulation partly as intact di- and tripeptides, largely through PEPT1/PEPT2. Triple-helical collagen and larger collagen fragments are recognized in vitro by integrins (α2β1), discoidin domain receptors (DDR2), and the immune receptors LAIR-1 and GPVI through repetitive Gly-Pro-Hyp motifs. A third point remains open: whether the small absorbed peptides engage those same receptors at attainable plasma concentrations, or act principally through intracellular routes such as the Keap1–Nrf2 axis and through substrate provision. The translational statements below are constrained by that uncertainty. The present manuscript builds upon that mechanistic foundation to address the translational dimension of BCP science: what measurable, clinically relevant outcomes do these molecular interactions produce, and in which veterinary and biomedical contexts has the evidence crossed the threshold of rigorous validation?

**Figure 1:**
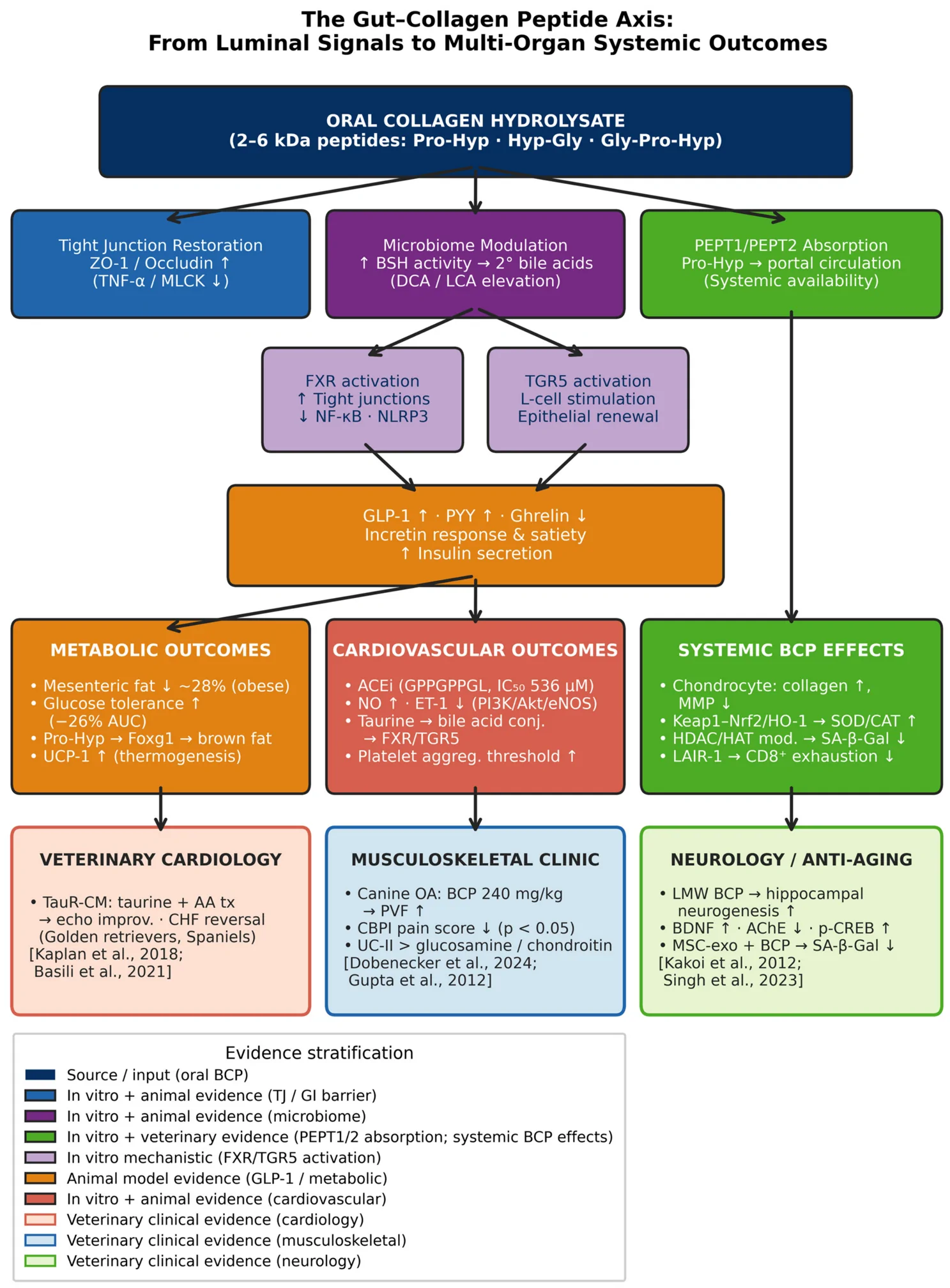
The gut–collagen peptide axis. Orally ingested collagen hydrolysate (2–6 kDa peptides) acts simultaneously at three levels: (i) restoration of intestinal tight junctions via ZO-1/occludin upregulation; (ii) proposed microbiome modulation leading to secondary bile acid synthesis and FXR/TGR5 activation (hypothetical; see Section 2.2 and Section 2.5); and (iii) PEPT1/PEPT2-mediated systemic absorption of intact oligopeptides. Downstream organ-level outcomes are stratified by evidence level (color-coded) (Kaplan et al., 2018; Basili et al., 2021; Dobenecker et al., 2024; Gupta et al., 2012; Kakoi et al., 2012; Singh et al., 2023) [2,3,4,5,6,7]. In the diagram, solid black arrows between blocks denote the directional flow of luminal signaling, systemic absorption, and downstream physiological cascades; within the text boxes, upward arrows (↑) indicate upregulation, activation, or clinical elevation, whereas downward arrows (↓) represent downregulation, inhibition, or clinical reduction. Abbreviations: BCP, bioactive collagen peptide; ZO-1, zonula occludens-1; MLCK, myosin light chain kinase; TNF-α, tumor necrosis factor-alpha; BSH, bile salt hydrolase; DCA, deoxycholic acid; LCA, lithocholic acid; FXR, farnesoid X receptor; TGR5, Takeda G-protein-coupled receptor 5; GPBAR1, G-protein-coupled bile acid receptor 1; GLP-1, glucagon-like peptide-1; PYY, peptide YY; PEPT1/2, peptide transporter 1/2; Pro-Hyp, prolyl-hydroxyproline; Foxg1, forkhead box G1; PGC-1α, peroxisome proliferator-activated receptor-gamma coactivator 1-alpha; UCP-1, uncoupling protein 1; ACE, angiotensin-converting enzyme; NO, nitric oxide; ET-1, endothelin-1; eNOS, endothelial nitric oxide synthase; Keap1, Kelch-like ECH-associated protein 1; Nrf2, nuclear factor erythroid 2-related factor 2; HO-1, heme oxygenase-1; SA-β-Gal, senescence-associated beta-galactosidase; LAIR-1, leukocyte-associated immunoglobulin-like receptor 1; MMP, matrix metalloproteinase; BDNF, brain-derived neurotrophic factor.

The gastrointestinal tract serves a dual physiological role. It is simultaneously the site of BCP absorption and a primary target organ where luminal peptide fragments exert independent physiological influence on the intestinal epithelium, the host microbiome, and the enteroendocrine system. This network—herein termed the gut–collagen peptide axis—is a working model connecting dietary protein composition to systemic metabolic, immune, and endocrine signaling through bile acid (BA) receptor activation. It is assembled from separate lines of evidence, and the causal chain has not been demonstrated end-to-end in any species. The model is offered to organize effects that extend beyond the musculoskeletal system for which collagen has traditionally been prescribed, not as a demonstrated pathway. A schematic overview of this axis is provided in Figure 1.

To maintain scientific rigor, all evidence presented in this review is explicitly stratified by its methodological origin: in vitro mechanistic data, controlled animal model studies, or validated veterinary or human clinical trials. This distinction is particularly important in veterinary medicine, where the translational gap between rodent biochemistry and large-companion-animal clinical outcomes is frequently underappreciated.

## 2. The Gut–Collagen Peptide Axis

### 2.1. Restoration of Intestinal Tight Junctions

The gastrointestinal tract serves not merely as the site of peptide absorption but as a primary target organ where collagen fragments exert substantial physiological influence [8]. Intestinal permeability—initiated by weaning stress, dietary toxins, heat stress, or lipopolysaccharide (LPS) challenge from pathogenic bacteria—triggers systemic endotoxemia and sustains chronic, low-grade inflammation. Resistance to this challenge depends on the structural integrity of the apical tight junction network, which is composed of claudins, occludin, and zonula occludens-1 (ZO-1).

Collagen peptides, particularly sequences rich in glutamine and hydrophobic residues, function as mucosal cytoprotectants. In Caco-2 cell monolayers, 24 h pre-incubation with a 3 kDa permeate fraction of Alaska pollock skin collagen hydrolysate (2 mg/mL), and in vivo animal models of stress-induced colitis and bacterial enteritis, have demonstrated that exposure to specific CH rescues the expression and correct spatial distribution of ZO-1 and occludin [8]. This restoration is mechanistically driven by the attenuation of tumor necrosis factor-alpha (TNF-α) signaling. Through modulation of the myosin light chain kinase (MLCK) pathway, BCPs prevent the cytokine-driven phosphorylation of the myosin regulatory light chain, thereby halting the perijunctional actomyosin ring contraction that otherwise forces tight junctions open. Murine chronic colitis models further confirm that collagen peptide-enriched diets restore both histological mucosal architecture and the hypoxia-inducible factor 1-alpha (HIF1α) dependent antioxidative pathway [9].

All of this evidence comes from Caco-2 monolayers and from chemically induced rodent colitis. No study has examined collagen hydrolysate in naturally occurring canine or feline chronic enteropathy, and intestinal permeability has never been measured in a companion animal after collagen supplementation. In equine practice the clinical weight of intestinal disease is considerable: in a multicentre study of 451 horses undergoing colic surgery, short-term survival was 68.5%, and packed cell volume at admission, post-surgical total plasma protein, and body condition score carried the greatest prognostic weight [10]. Whether nutritional support of barrier function alters such outcomes has not been tested; the study is cited here only to indicate the clinical burden against which any such intervention would have to be measured.

### 2.2. Microbiota Modulation, Bile Acid Receptor Signaling (FXR/TGR5), and Dietary Fiber Interactions

A central mechanism underlying collagen peptide efficacy involves the modulation of the host microbiome and the subsequent alteration of BA metabolism. Bile acids are not merely digestive detergents; they are potent endocrine signaling molecules dynamically transformed by the gut microbiota into secondary BAs, including lithocholic acid (LCA), deoxycholic acid (DCA), and ursodeoxycholic acid (UDCA). The diagnostic relevance and analytical complexity of BA profiling in canine patients—including the matrix-specific, breed-dependent, and diet-sensitive nature of BA measurement—have been comprehensively reviewed [11] and elaborated in the context of canine clinical practice by [12,13]. A recent exploratory clinical study reports that fasting serum bile acid profiles—a cholic acid-dominated elevation of unconjugated primary species and a monotonic increase in the primary-to-secondary ratio—differed between healthy controls and dogs with food-responsive or immunosuppressant-responsive enteropathy [14]. The study was small and used unmatched, younger purpose-bred controls, so these findings identify a candidate serum pattern rather than a diagnostic or treatment-predictive marker.

In a diet-induced obese mouse model, low-molecular-weight collagen peptides (1 mg/mouse/day for 10 weeks) raised faecal microbial alpha diversity relative to the high-fat control and shifted the relative abundance of several genera [9,15]. This microbial shift increases the biosynthesis of secondary BAs, which are potent agonists of the Takeda G-protein-coupled receptor 5 (TGR5, also known as GPBAR1); the farnesoid X receptor (FXR) is engaged most strongly by the primary bile acid chenodeoxycholic acid, with secondary bile acids acting as weaker FXR ligands. Activation of intestinal FXR enforces mucosal barrier integrity—directly inducing tight junction gene transcription—reduces lipogenesis, and suppresses mucosal pro-inflammatory NF-κB and NLRP3 inflammasome signaling, substantially mitigating inflammatory bowel disease (IBD) and colitis [16,17]. Concurrently, TGR5 activation on enteroendocrine L-cells and Kupffer cells promotes epithelial renewal, protects microglia in neuroinflammatory models, and influences systemic energy metabolism. This is a compositional association. Bile salt hydrolase (BSH) activity was not assayed in that study, the abundance of BSH-carrying taxa was not quantified, and faecal or serum bile acids were not measured; the link from the microbial shift to the bile acid pool is inferred from the known bile salt hydrolase capacity of the genera involved rather than demonstrated in the collagen peptide literature. No study has shown that the microbial shift is necessary for the systemic effects reported.

Taurine conjugation provides a second, mechanistically independent input to the same enterohepatic bile acid pool. Taurine is the principal conjugation substrate for primary BAs in carnivorous species, and impaired mitochondrial tRNA taurine modification leads to defective taurocholate synthesis, disrupted BA homeostasis, and secondary FXR/TGR5 signaling insufficiency, as shown in taurine-depleted feline models [18,19]. Taurine status does not derive from collagen intake: taurine is synthesized from the sulfur amino acids cysteine and methionine, which collagen and gelatin are essentially devoid of [20]. Collagen peptides and taurine therefore act as parallel dietary levers that converge on the bile acid pool—collagen through microbial secondary BA production, taurine through conjugation capacity—rather than as a single collagen-driven pathway. This distinction is clinically relevant in carnivore nutrition: high-collagen or gelatin-rich diets supply abundant glycine and proline but no taurine precursors, and cannot offset inadequate dietary taurine or cysteine.

A complementary dietary dimension is provided by soluble fiber, which modulates BA kinetics through a distinct mechanical sequestration mechanism. A prospective within-subject study employing high-sensitivity LC-MS/MS profiling—validated for canine BA measurement [21]—demonstrated that psyllium supplementation (0.2 g/kg body weight for 14 days) in healthy dogs significantly increased total serum BA exposure (AUC +24.8%; *p* < 0.05) and drove a +33.3% rise in the primary BA fraction, while secondary BA concentrations remained nearly stable, markedly elevating the primary-to-secondary bile acid ratio (*p* < 0.001) [22]. This response reflects increased hepatic synthesis rather than microbial biotransformation—opposite in directionality to BCP-driven effects—and is mechanistically consistent with FXR signaling modulation documented in murine colitis models [23]. Psyllium and collagen hydrolysate have never been co-administered in a controlled study. The psyllium data stand on their own as an established finding in healthy dogs; any interaction with collagen hydrolysate—additive, neutral, or opposing—is speculation, and is noted here only because concurrent dietary interventions confound the attribution of systemic effects to either agent. A recent framework for multi-factorial disruption of enterohepatic bile acid homeostasis in companion animals [24] situates these effects within a broader systemic network; within that framework, the gut–collagen peptide axis can be regarded as one of several nutritional levers modulating the BA pool.

The sequence proposed in this section—collagen peptides, then a microbial shift, then increased secondary BAs, then FXR and TGR5 activation, then systemic metabolic and immune effects—has not been demonstrated as a causal chain. Each link rests on a separate experiment in a different model. No study has used receptor antagonism, germ-free or antibiotic-treated animals, or bile acid sequestration to test whether the downstream effects depend on the upstream step, and no study has measured bile acid receptor occupancy after collagen supplementation in any species. The sequence should be read as a hypothesis to be tested.

### 2.3. Endocrine Regulation: GLP-1, PYY, and Ghrelin

Two routes can raise enteroendocrine GLP-1 and PYY after collagen ingestion, and the available data do not separate them. TGR5 on enteroendocrine L-cells is activated by secondary bile acids and signals through cAMP to promote GLP-1 and PYY release [25]. In parallel, a 15–20 g protein or gelatin load is itself a direct secretagogue: luminal amino acids and peptides stimulate L-cells independently of bile acids. The collagen studies below report the incretin output but do not isolate the TGR5-mediated component, so the bile acid route remains a hypothesis rather than a demonstrated mechanism. Once released, GLP-1 and PYY slow gastric emptying, increase glucose-dependent insulin secretion, and act on the central nervous system to suppress appetite and induce satiety.

Human randomized controlled trials demonstrate that seven days of collagen peptide supplementation (15 g/day), with the final dose consumed post-exercise, significantly elevated plasma GLP-1 area under the curve (AUC) compared to placebo (CON: 6369 vs. CP: 9064 pmol/L; *p* < 0.001), and reduced both ghrelin and leptin concentrations in physically active females [26]. Earlier human data confirm that a single 20 g gelatin meal produces a significant GLP-1 rise peaking at 60–120 min post-ingestion in both normal-weight and obese subjects, accompanied by a corresponding insulin increase [27]. In C57BL/6 diet-induced obese mice, eight weeks of low-molecular-weight bovine collagen peptide supplementation reduced mesenteric, visceral, and total adipose tissue (by approximately 28%, 15%, and 18%, respectively) and improved glucose tolerance (−26% in the intraperitoneal glucose tolerance test area under the curve); a human randomized controlled trial of a low-digestibility, high-swelling-capacity collagen reported parallel anti-obesity effects [15,28]. None of these studies measured bile acid changes or blocked TGR5; they establish that collagen supplementation raises GLP-1 and reduces adiposity, but not that the effect is bile acid-mediated.

### 2.4. Pro-Hyp-Driven Brown Adipocyte Differentiation: An In Vitro Mechanism with Unconfirmed In Vivo Relevance

A mechanistically distinct contribution of BCPs to metabolic homeostasis was identified by Nomura and colleagues (2024), who investigated the role of the signature collagen dipeptide prolyl-hydroxyproline (Pro-Hyp) in adipocyte biology. In C3H10T1/2 mesenchymal stem cells treated with rosiglitazone, Pro-Hyp treatment decreased adipocyte size and upregulated brown fat-specific genes, including C/EBPα, PGC-1α, and UCP-1, without altering PPARγ expression [29]. A Pro-Hyp responsive element was identified in the PGC-1α gene promoter, which facilitated the binding of the Foxg1 transcription factor—a regulatory mechanism not previously described in collagen biology. Pro-Hyp also elevated mitochondrial activity, consistent with enhanced brown adipocyte thermogenic function [29].

The experiments were performed in C3H10T1/2 cells. Whether Pro-Hyp reaches brown or beige adipose tissue at comparable concentrations after oral dosing, and whether it alters energy expenditure in a living animal, has not been tested. The combination of incretin-mediated intake suppression and Pro-Hyp-mediated expenditure is a hypothesis; no anti-obesity claim for collagen supplementation in dogs, cats, or horses is supported by current data [30]. Together, the GLP-1 incretin response with ghrelin suppression and the Pro-Hyp/Foxg1/PGC-1α brown adipogenesis pathway represent two candidate, mechanistically independent arms of BCP-mediated metabolic regulation, neither of which has been confirmed in a target species.

### 2.5. What the Gut–Collagen Peptide Axis Does and Does Not Establish

The elements of this axis are not supported by evidence of equal strength, and the sections above should be read with that gradient in mind.

Three observations rest on direct experiment. Collagen peptides restore ZO-1 and occludin expression and localisation in Caco-2 monolayers and in rodent colitis, with suppression of TNF-α-driven MLCK signaling. A protein or gelatin load of 15–20 g raises circulating GLP-1 and PYY in human subjects. Pro-Hyp raises PGC-1α, UCP-1, and C/EBPα expression in C3H10T1/2 cells through a Foxg1-dependent promoter element.

One observation is an association. Collagen peptide feeding changes the composition of the rodent intestinal microbiome. This has not been shown to be necessary for any downstream outcome.

Five propositions remain hypothetical. That the microbial compositional shift specifically increases the abundance of bile-salt-hydrolase-carrying taxa, which was not quantified in the cited study. That this in turn raises secondary bile acid synthesis, which was not measured. That the microbial shift causes the change in the bile acid pool. That the changed bile acid pool activates FXR or TGR5 to a physiologically meaningful degree. That this activation, rather than direct amino acid stimulation of enteroendocrine cells or direct substrate provision, produces the metabolic, endocrine, and immune effects attributed to collagen supplementation. No study has attempted the experiments that would separate these possibilities—receptor antagonism, germ-free animals, bile acid sequestration, or measurement of receptor occupancy. Table 1 summarizes the position mechanism by mechanism.

## 3. Musculoskeletal Therapeutic Applications

### 3.1. Canine Osteoarthritis: Biomechanical Restoration and Clinical Trials

The clinical use of collagen hydrolysate in joint disease predates the mechanistic literature reviewed here: absorption, cartilage accumulation, and the early clinical data were first brought together two decades ago [31], on the basis of four open-label and three double-blind human studies of variable methodological quality.

Osteoarthritis (OA) is the most prevalent chronic degenerative joint disease in the canine population, characterized by progressive destruction of articular cartilage, subchondral bone sclerosis, and synovial inflammation. Traditional pharmacological management relies heavily on non-steroidal anti-inflammatory drugs (NSAIDs) and corticosteroids, which provide symptomatic relief but carry significant risks of gastrointestinal, hepatic, and renal toxicity upon long-term administration. BCPs represent a well-supported nutraceutical intervention that targets the structural pathophysiology of OA rather than merely masking its symptomatology [32].

A seminal 2024 double-blind, placebo-controlled clinical trial evaluated the efficacy of specific BCPs in dogs with naturally occurring OA using objective force-plate kinetics on a horizontal treadmill fitted with four integrated piezoelectric force plates. The design was a double-blind, placebo-controlled, three-arm field study; 31 of 41 enrolled dogs completed it. Over twelve weeks the groups received specific bioactive collagen peptides (240 ± 95 mg/kg body weight per day), omega-3 fatty acids with vitamin E (700 ± 115 mg/kg plus vitamin E), or a cellulose placebo. Only the collagen peptide group improved: peak vertical force (PVF) of the affected limb rose significantly (Δ PVF, *p* = 0.015), the relative PVF gain was highly significant within the group (*p* < 0.001) and significant against placebo (*p* = 0.012), vertical impulse increased (Δ VI, *p* = 0.004), and the PVF symmetry index improved (*p* = 0.041). The placebo group deteriorated over the same period, as expected in a progressive disease. No NSAID comparator was included, so no statement about relative adverse-event rates can be drawn from this trial [4]. The owner-assessed outcomes were more limited. Quality of life, measured within the CBPI, improved in the collagen peptide group by 54.6 ± 73.3% relative to baseline, significantly more than in the omega-3 group (−2.5 ± 62.1%; *p* = 0.015); the difference against placebo (25.0 ± 40.7%) was not significant (*p* = 0.517), and placebo itself produced a significant within-group improvement. The CBPI pain score showed no systematic difference between the three groups, and accelerometry detected no change in daily activity. The objective kinetic gain is therefore the finding that carries the evidence; a caregiver placebo effect is measurable in this design, and a subjective analgesic benefit is not established [4].

Three uncertainties remain. The 240 mg/kg regimen has not been compared with a lower or a higher dose in any controlled canine trial, so the dose–response relationship is unknown and the reported dose should not be read as an optimum. Controlled trials do not exceed twelve weeks, and no study has followed treated dogs beyond that point; whether the kinetic gain persists, plateaus, or regresses has not been examined. And no trial has compared collagen hydrolysate against an NSAID at an equivalent analgesic endpoint, so its clinical positioning—adjunct, NSAID-sparing agent, or standalone therapy—is undefined. The trial was funded by a collagen manufacturer, and one co-author is a co-inventor on collagen peptide patents.

**Figure 2:**
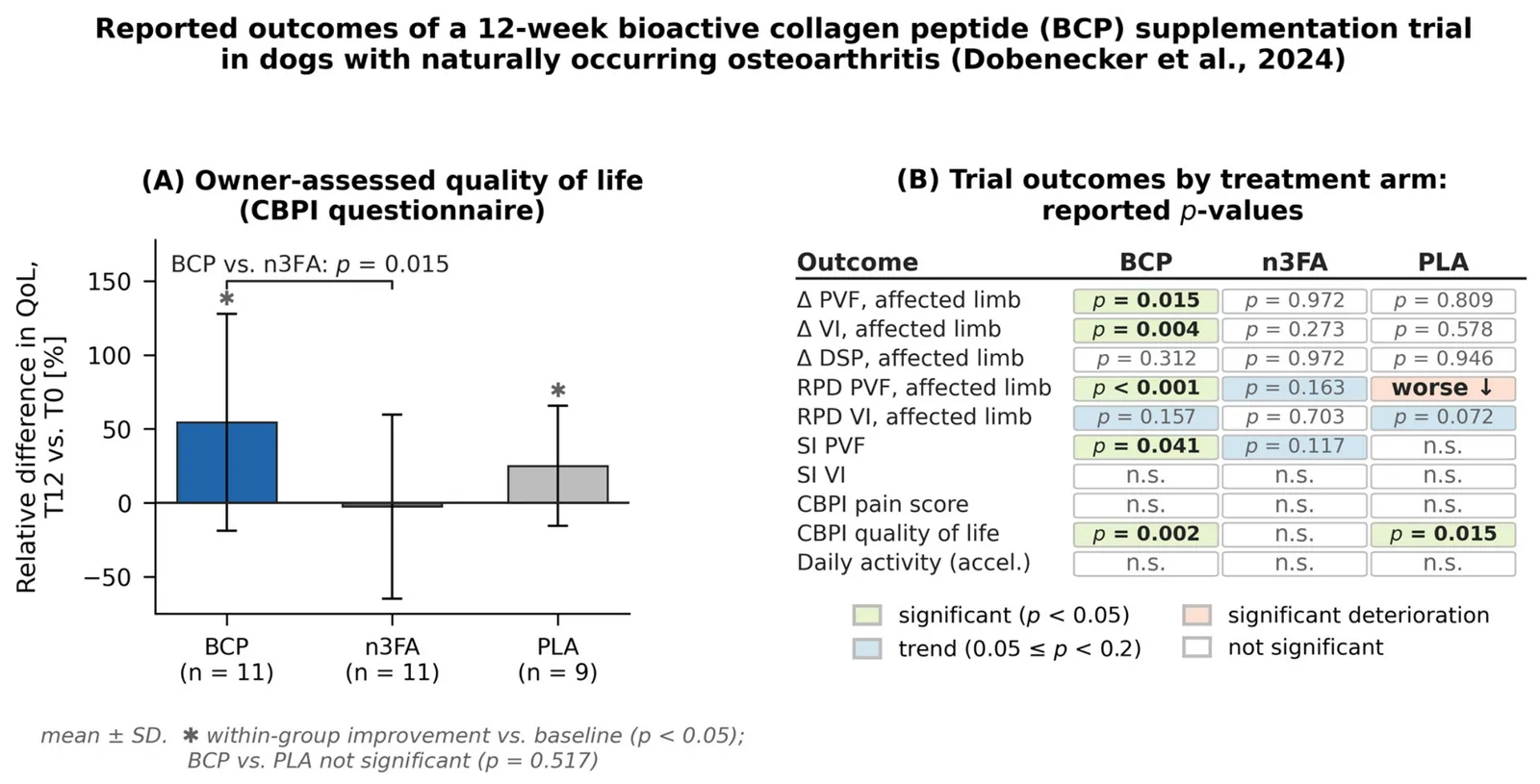
Reported outcomes (**A**) Owner-assessed quality of life, expressed as the relative difference between week 12 and baseline; bars show mean ± SD, and the values above each bar are within-group tests against the zero-change hypothesis. (**B**) All reported trial outcomes by treatment arm, with the *p*-value given for each; cell shading distinguishes significant results, trends, and significant deterioration. Data from Dobenecker et al. (2024) [4]. In panel (**A**), bar colors serve strictly for visual differentiation of the treatment arms. In panel (**B**), cell shading reflects the statistical and clinical interpretation of the outcomes: light green indicates statistically significant improvement (*p* < 0.05), light blue denotes a positive statistical trend (0.05 ≤ *p* < 0.2), light red represents significant clinical deterioration (indicated also by the downward arrow ↓), and white designates non-significant results (n.s.). Abbreviations: BCP, bioactive collagen peptide; n3FA, omega-3 fatty acids with vitamin E; PLA, placebo; QoL, quality of life; CBPI, Canine Brief Pain Inventory; PVF, peak vertical force; VI, vertical impulse; DSP, duration of stance phase; SI, symmetry index; Δ, absolute difference between week 12 and baseline; RPD, relative difference; SD, standard deviation; n.s., not significant.

The clinical resolution of lameness is mechanistically underpinned by the accumulation of BCPs within the articular matrix, where they stimulate chondrocytes to upregulate the de novo biosynthesis of type II collagen and aggrecan, while simultaneously suppressing pro-catabolic interleukins and matrix metalloproteinases (MMPs) [33,34]. The objective kinetic and pain outcome data from canine OA trials are illustrated in Figure 2.

### 3.2. Undenatured Type II Collagen (UC-II) as an Immunological Alternative

A mechanistically distinct approach to joint management employs undenatured type II collagen (UC-II), which operates through oral immune tolerance induction rather than direct matrix substrate provision. UC-II contains intact triple-helical epitopes that, upon interaction with gut-associated lymphoid tissue (GALT) in the Peyer’s patches, stimulate regulatory T cells (Tregs) to suppress autoimmune cartilage attack via transforming growth factor-beta (TGF-β) and interleukin-10 (IL-10) secretion.

A comparative veterinary clinical trial in arthritic dogs demonstrated that UC-II (10 mg/day) produced significantly greater reductions in ground-force-plate-measured lameness scores than a combination of glucosamine (2000 mg/day) plus chondroitin (1600 mg/day) at equivalent dosing intervals [5]. A subsequent equine study confirmed superior efficacy of UC-II over glucosamine/chondroitin in horses with OA [35]. UC-II acts through a mechanism orthogonal to that of collagen hydrolysate. No controlled trial has compared the combination with either agent alone, and orthogonal mechanisms do not guarantee an additive clinical effect. The combined protocol is untested.

### 3.3. Equine Osteoarthritis and Tendinopathy

In horses, a two-centre pilot study of 38 privately owned animals with mild to moderate naturally occurring OA reported improvement after twelve weeks of oral BCP (PETAGILE) at 25 g or 50 g per day. Attending veterinarians scored eight orthopaedic parameters; in the 50 g group a strong effect size (Cohen’s r > 0.5) was reached in six of eight, with lameness and flexion pain already improved at six weeks. The assessment rested on veterinary clinical scoring and an owner questionnaire, not on force-plate kinetics; only one of the two centres included a placebo arm, and the authors themselves call for blinded, placebo-controlled confirmation. The supplement was supplied by the manufacturer [36]. The underlying pharmacological rationale parallels the canine data: Pro-Hyp and Hyp-Gly accumulate in joint tissues after oral administration, directly stimulating equine chondrocyte and synoviocyte matrix production while attenuating pro-inflammatory mediator synthesis [1].

Particularly relevant to equine sports medicine is the evidence for pre-exercise vitamin C co-administration with gelatin-based collagen hydrolysate. A randomized controlled human trial demonstrated that 15 g of gelatin consumed with 48 mg of vitamin C one hour prior to intermittent activity doubled the collagen synthesis response, as measured by circulating amino-terminal propeptide of type I collagen (P1NP) [37,38].

That trial was conducted in human volunteers. Unlike humans, horses synthesize ascorbate endogenously, so the rationale for exogenous vitamin C co-administration does not transfer directly across the species boundary. Pre-exercise gelatin with vitamin C is at most a translational hypothesis for equine use: it has not been tested in horses, and it is not an evidence-based recommendation.

### 3.4. Feline Osteoarthritis and Mobility

Feline osteoarthritis is notoriously underdiagnosed due to the species’ evolutionary predisposition to mask pain through behavioral adaptation rather than overt lameness. Radiographic evidence suggests OA affects a substantial proportion of the general feline population and more than 90% of cats older than 12 years [39].

In a veterinary trial, nutraceutical supplements—including glucosamine/chondroitin sulfate combinations—demonstrated measurable improvements in activity and owner-assessed mobility in cats with degenerative joint disease [40]. Collagen hydrolysate-specific randomized controlled trials in cats remain absent, representing a significant translational gap. The high tolerability and absence of gastrointestinal side effects make oral liquid or powder BCP formulations particularly suitable for long-term chronic pain management in geriatric felines, where polypharmacy and NSAID-associated nephrotoxicity are primary clinical concerns.

### 3.5. Livestock Production: Growth Performance and Connective Tissue Integrity

In commercial swine and poultry production, the endogenous synthesis of glycine, proline, and hydroxyproline is frequently inadequate to support the genetic potential for rapid skeletal and muscular hypertrophy. The accelerated accumulation of muscle mass outpaces connective tissue scaffolding synthesis, leading to widespread locomotor disorders, subclinical tendinopathies, poor bone mineral density, and diminished meat quality [41].

High-quality systematic controlled trials in commercial livestock remain limited, and extrapolation from rodent and companion animal data must therefore be interpreted with appropriate caution. Currently, no high-evidence randomized controlled trials of bioactive collagen peptides are available in livestock species.

## 4. Cardiovascular Applications and Amino Acid Deficiency Syndromes

### 4.1. Taurine-Responsive Dilated Cardiomyopathy (TauR-CM)

A clinically critical and potentially reversible manifestation of dietary amino acid insufficiency in veterinary cardiology is taurine-responsive dilated cardiomyopathy (TauR-CM), first described in cats [42] and subsequently identified in multiple canine breeds [2,43,44]. The connection to the enterohepatic bile acid axis runs through conjugation rather than through collagen: taurine serves as the principal conjugation substrate for primary BAs in obligate carnivores [19], and impaired mitochondrial tRNA taurine modification results in defective taurocholate synthesis and disrupted enterohepatic bile acid homeostasis [18]; in parallel, taurine deficiency compromises cardiomyocyte mitochondrial stability, linking the conjugation defect to myocardial dysfunction. The broader significance of mitochondrial dysfunction—extending beyond cardiomyopathy to connective tissue integrity and metabolic diseases in livestock—has been elaborated by [45], providing an important interspecies framework for interpreting mitochondrial taurine insufficiency.

In a multicentre prospective observational study of golden retrievers, 23 of 24 dogs with taurine deficiency and dilated cardiomyopathy (DCM) were fed diets that were grain-free, legume-rich, or both. After diet change and taurine supplementation, 23 of 24 showed improvement in echocardiographic parameters and normalisation of taurine concentrations, and congestion resolved in 9 of 11 dogs that had presented in congestive heart failure (CHF). The design is observational and uncontrolled, and the authors describe the aetiology as multifactorial—dietary, metabolic, and genetic; a causal role for diet cannot be assigned from these data, although the reported reversal following taurine repletion is a strong clinical signal [2]. Subsequent investigations confirmed the same syndrome in English cocker spaniels consuming diets with low cysteine content [3]. The broader debate over a causal role of diet in canine DCM has been reviewed elsewhere [46]; a subsequently published corrigendum discloses that the review’s authors are affiliated with an organization providing nutritional consulting to pet-food manufacturers, including producers of grain-free, pulse-rich diets [46]. The diagnostic evaluation of TauR-CM cases should incorporate BA profiling alongside standard taurine measurement, given that BA conjugation status serves as a functional readout of taurine metabolic sufficiency; validated LC-MS/MS methods for canine BA analysis are available for this purpose [11,21].

Nothing in this section implies that collagen supplementation affects taurine status. Collagen and gelatin contain essentially no cysteine or methionine and therefore supply no taurine precursors [20]. Taurine and collagen peptides enter the BA pool by separate routes—conjugation capacity and microbial secondary bile acid production, respectively—and a collagen-rich diet cannot correct a taurine deficiency. The two subjects are treated together in this review because they converge on one compartment, not because they are alternatives [2].

**Figure 3:**
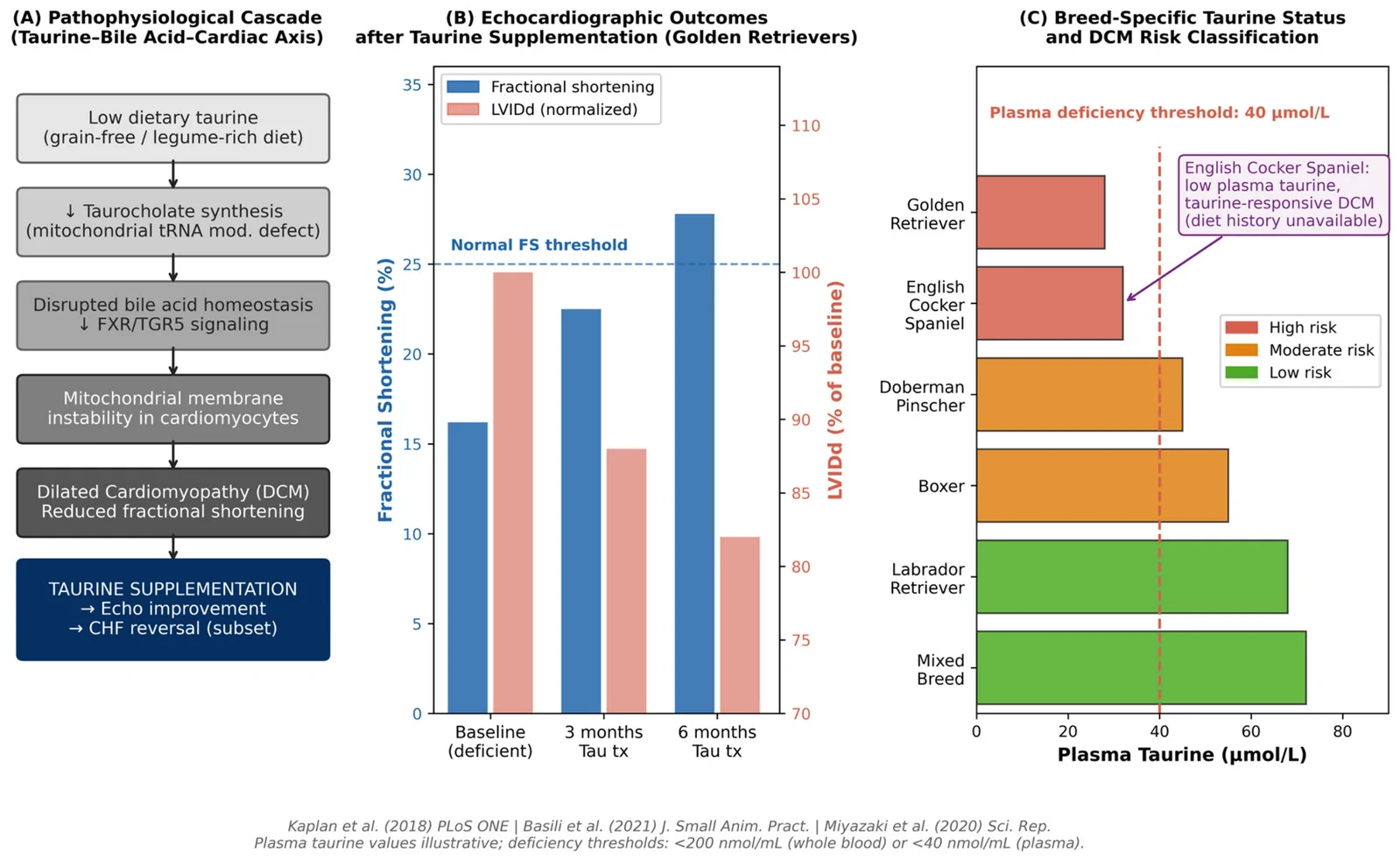
Taurine-responsive dilated cardiomyopathy (TauR-CM): pathophysiology, echocardiographic outcomes, and breed risk classification. (**A**) Pathophysiological cascade from low dietary taurine intake to dilated cardiomyopathy and therapeutic reversal. (**B**) Illustrative echocardiographic improvements in fractional shortening (%) and normalized left ventricular internal diameter in diastole (LVIDd) following taurine supplementation in golden retrievers. (**C**) Breed-specific plasma taurine concentrations and DCM risk stratification (Kaplan et al., 2018; Basili et al., 2021; Miyazaki et al., 2020) [2,3,18]. In panel (A), box colors serve strictly for visual differentiation between the progressive pathological cascade (grey shading) and the therapeutic intervention (blue box), whereas solid black arrows indicate the sequential direction of the pathophysiological mechanism. Abbreviations: TauR-CM, taurine-responsive dilated cardiomyopathy; DCM, dilated cardiomyopathy; CHF, congestive heart failure; FXR, farnesoid X receptor; TGR5, Takeda G-protein-coupled receptor 5; FS, fractional shortening; LVIDd, left ventricular internal diameter in diastole; µmol/L, micromole per litre.

The pathophysiological cascade, echocardiographic treatment response, and breed-specific taurine risk classification are illustrated in Figure 3. Species-specific and breed-specific taurine reference ranges, deficiency thresholds, and TauR-CM risk classifications are summarized in Table 2. In Figure 3, box shading in panel (A) denotes severity progression only, and the dashed line in panel (C) marks the deficiency threshold at 40 µmol/L; the data in panel (B) are illustrative, not measurements reproduced from the original report.

### 4.2. Taurine and Platelet Function: Evidence from Metabolic Disease

The cardiovascular protective potential of taurine extends beyond myocardial stabilization to the regulation of platelet aggregation and endothelial function. In human subjects with insulin-dependent diabetes mellitus (IDDM), plasma and platelet taurine concentrations were significantly lower than in matched controls, and this depletion was associated with a significantly reduced threshold for arachidonic acid-induced platelet aggregation [51]. Oral taurine supplementation (1.5 g/day for 90 days) normalized both plasma and platelet taurine levels in diabetic patients and restored platelet aggregation threshold to values equivalent to healthy controls [52]. In vitro, taurine reduced platelet aggregation in diabetic patients in a dose-dependent manner, while having no effect on aggregation in healthy subjects—confirming a pathology-specific modulatory role [51].

These data come from human patients with insulin-dependent diabetes. No comparable study exists in dogs or cats, platelet taurine has not been evaluated as a therapeutic target in veterinary medicine, and taurine status is not a readout of collagen intake. This section describes a taurine-specific effect in a human metabolic disease; it is not evidence of an antiplatelet action of collagen supplementation [51,52].

### 4.3. ACE-Inhibitory Collagen Peptides and Endothelial Protection

Beyond taurine-mediated mechanisms, targeted collagen peptides manifest direct antihypertensive bioactivity through competitive inhibition of the angiotensin-I-converting enzyme (ACE) [53]. Marine-derived collagen peptides—specifically sequences extracted from jellyfish (*Chrysaora* sp.), *Takifugu bimaculatus* skin, and eel bone (*Anguilla japonica*)—possess structural motifs that insert deeply into the S2 active pocket of the ACE molecule, neutralizing its catalytic potential via hydrogen bonding and electrostatic interactions. The sequence GPPGPPGL exhibits a half-maximal inhibitory concentration (IC50) of 535.84 µM under in vitro conditions [54].

In vitro assays demonstrate that administration of these sequences to angiotensin-II-stimulated human umbilical vein endothelial cells (HUVECs) significantly increases nitric oxide (NO) secretion while depressing endothelin-1 (ET-1) production, providing endothelial protection against apoptosis and oxidative stress via the PI3K/Akt/eNOS signaling pathway [54].

All of these data are in vitro: enzyme-inhibition assays and endothelial cell culture. The reported IC50 values lie in the hundreds of micromolar, whereas circulating collagen dipeptide concentrations after a standard oral dose are in the low micromolar range. No study in any species has demonstrated a blood-pressure effect of orally administered collagen peptides, and no veterinary antihypertensive claim is supported by the present evidence.

## 5. Hepatoprotection and Fibrosis Reversal

One distinction runs through this section. Collagen deposited by activated hepatic stellate cells, and collagen deposited by cancer-associated fibroblasts, are pathological products of the host. Orally administered collagen peptides are an exogenous nutritional input. The two are connected through a shared molecular vocabulary and not through a demonstrated physiological relationship. No study has shown that dietary collagen peptides accelerate, retard, or otherwise modify hepatic fibrosis or tumour stroma in vivo.

### 5.1. Hepatic Stellate Cell Activation and Fibrotic Cascade

Non-alcoholic fatty liver disease (NAFLD) and chronic liver fibrosis represent major disease burdens driven by the aberrant activation of hepatic stellate cells (HSCs). Under conditions of chronic hepatic injury or metabolic overload, HSCs located in the perisinusoidal space of Disse transdifferentiate from quiescent, vitamin-A-storing cells into aggressive, proliferative myofibroblasts [55]. These activated cells deposit massive amounts of type I and type III collagen, driving the progression from non-alcoholic steatohepatitis (NASH) to cirrhosis.

Glycine, the most abundant amino acid in collagen peptides, is one of the three constituent amino acids of glutathione and can become a co-limiting substrate for hepatic glutathione (GSH) synthesis under conditions of high oxidative demand; the supporting rodent data are detailed in Part I (Section 3.4) [56,57,58]. In the hepatic context this links collagen-derived glycine supply to the antioxidant capacity that constrains stellate cell activation. This is a substrate-level inference drawn from rodent glycine supplementation. Collagen hydrolysate itself has not been tested in a model of hepatic fibrosis in any veterinary species.

### 5.2. Oncological Stroma and LAIR-1-Mediated Immunomodulation

Cancer-associated fibroblasts (CAFs) in solid tumors exploit collagen synthesis pathways to construct a desmoplastic stroma that physically excludes immune effector cells and promotes drug resistance [59]. Concurrently, the repetitive Gly-Pro-Hyp motifs of tumor stromal collagen engage LAIR-1 on tumor-infiltrating immune cells, inducing CD8+ T cell exhaustion via the SHP-1/SHP-2 and CAMK1-CREB signaling pathways [1,60]. From a veterinary oncology perspective, this axis is of particular relevance to canine mammary carcinoma, where increased stromal collagen density and fiber organization are documented negative prognostic factors [61]; analogous tumor–stromal interactions are of emerging interest in other companion-animal solid tumors. This concerns tumour-derived collagen acting as an immunosuppressive ligand. It carries no implication, in either di-rection, for oral collagen peptide supplementation in tumour-bearing animals, which has not been studied.

## 6. Neurological Repair and Behavioral Neuroscience

### 6.1. Neuroprotection and CNS Repair in Animal Models

Recent studies demonstrate the capacity of collagen peptides to influence central nervous system (CNS) repair following systemic absorption. The extracellular matrix within the CNS is highly dynamic, relying on specific collagen isoforms to guide axonal maturation, stabilize synapses, and regulate astrocyte recruitment. In in vivo animal models of perinatal asphyxia—which produces long-term oxidative brain damage, spasticity, and cognitive deficits—the early intragastric administration of marine collagen peptides significantly prevents neurological decay [62]. The neuroprotective mechanism operates through a multi-factorial pathway: active scavenging of reactive oxygen species (ROS) within the cerebral cortex; downregulation of acetylcholinesterase (AChE) activity to enhance cholinergic signaling; and upregulation of brain-derived neurotrophic factor (BDNF) alongside phosphorylated cAMP-response element binding protein (p-CREB) in the hippocampus [62]. Perinatal asphyxia in rodents is remote from any veterinary clinical indication. The finding is a starting point for research and not a rationale for use.

### 6.2. Hippocampal Neurogenesis and Anxiolytic Effects

Kakoi and colleagues (2012) administered two enzymatically hydrolyzed collagen preparations—a lower-molecular-weight fraction (LP: below 2000 Da) and a higher-molecular-weight fraction (HP: approximately 30,000 Da)—to adult C57BL/6 mice for four weeks. LP administration produced a 1.2-fold increase in the density of proliferating cells in the subgranular zone of the hippocampus compared to HP. In elevated plus maze testing, LP mice also spent significantly less time in the closed arms, indicating reduced anxiety-related behavior [6]. In companion animals this is untested; it offers at most a preliminary rationale—not a clinical recommendation—for studying low- rather than high-molecular-weight formulations in behavioral indications.

### 6.3. Longevity and Anti-Senescence: Parallel Taurine and Collagen Peptide Contributions

Taurine declines with age across multiple mammalian species, and its supplementation has been shown to extend median lifespan and improve multiple markers of biological aging in mice, including bone density, muscle strength, and cognitive performance [7]. Taurine and collagen peptides contribute to healthy aging through separate mechanisms that can be considered together but should not be conflated. Collagen peptides act through two epigenetic routes: the modulation of HDACs and HATs, which reverses pathology-associated chromatin silencing in fibrotic and senescent cells [63]; and the Keap1–Nrf2/HO-1 activation pathway, wherein marine-derived BCP sequences activate the Keap1–Nrf2 axis to drive transcription of SOD, CAT, and HO-1 [64]. Synergy between BCPs and mesenchymal stem cell-derived extracellular vesicles (MSC-EVs) has been reported in vitro only, in cultures exposed to human umbilical cord MSC exosomes [65]. More broadly, MSC-derived EVs are reported to promote macrophage polarization toward an anti-inflammatory M2 phenotype and induce regulatory T-cell responses, mediated by EV-associated cytokines, growth factors, and regulatory microRNAs [66]; whether the same mechanisms underlie the anti-senescent BCP–MSC-EV synergy reported by Zhu et al. (2024) has not been examined [65].

None of the neurological or anti-senescence findings summarized in this section has been examined in a veterinary clinical population. Statements about neuroprotection, neurogenesis, behavioural improvement, and anti-aging describe rodent and cell-culture observations, and are reported here to define a research agenda rather than a therapeutic one.

## 7. Evidence Synthesis, Translational Limitations, and Future Directions

### 7.1. Summary of Therapeutic Applications

**Figure 4:**
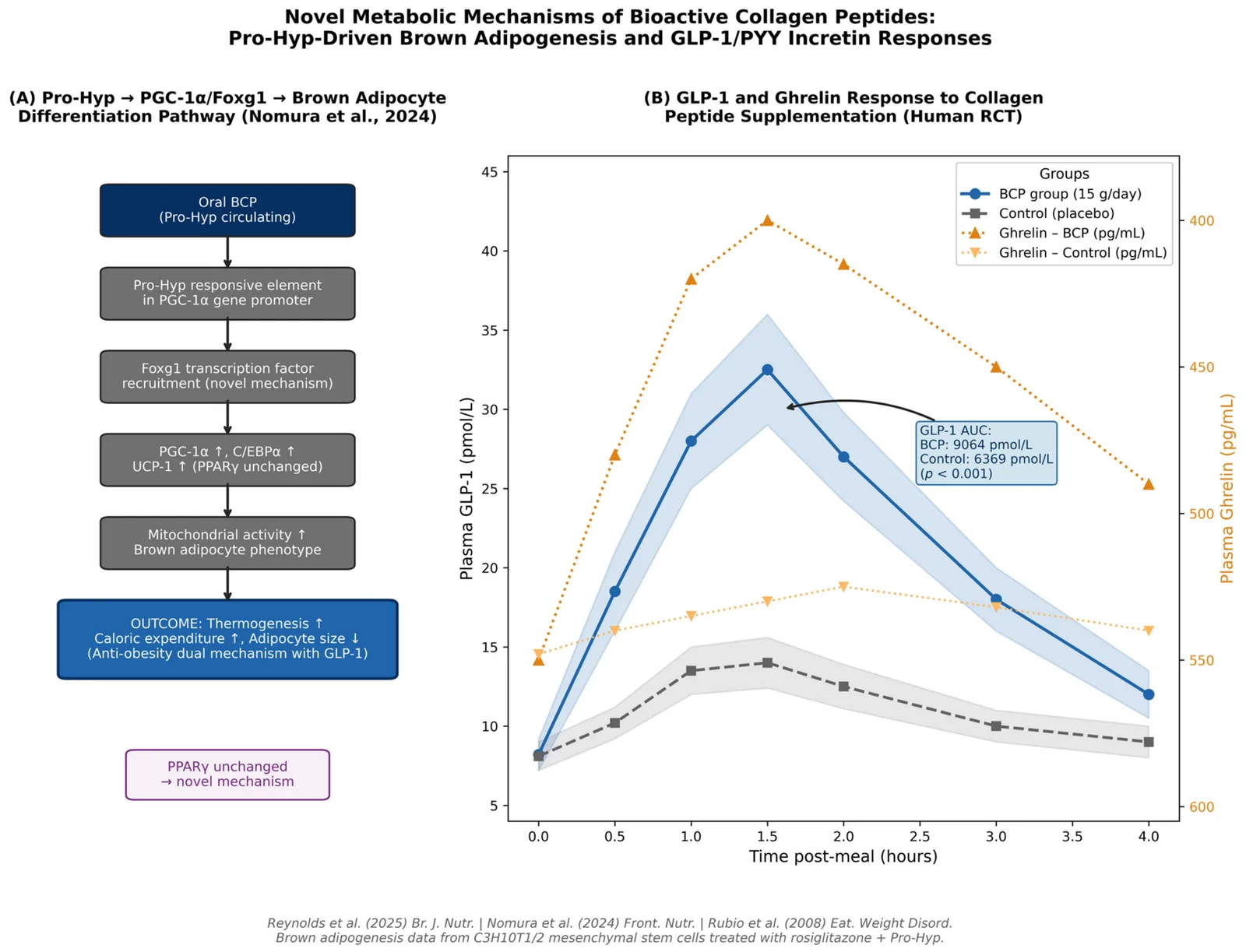
Novel metabolic mechanisms of bioactive collagen peptides. (**A**) Pro-Hyp → PGC-1α/Foxg1 → brown adipocyte differentiation pathway; PPARγ expression is not altered, confirming novelty of the Foxg1-mediated mechanism. (**B**) GLP-1 and ghrelin plasma kinetics following 15 g/day BCP supplementation versus placebo in physically active females (human RCT); secondary y-axis (ghrelin) is inverted. Shaded areas represent ±SEM (Reynolds et al., 2025; Nomura et al., 2024; Rubio et al., 2008) [26,27,29]. In panel (**A**), box colors serve strictly for visual differentiation between the initial trigger/terminal outcome (blue boxes) and the sequential mechanistic steps (grey boxes), whereas solid vertical arrows indicate the progressive direction of the signaling pathway. Within the text boxes, upward arrows (↑) denote upregulation or enzymatic activation, and downward arrows (↓) represent cellular size reduction. In panel (**B**), line colors and symbols distinguish experimental groups and target hormones, with the curved arrow linking the GLP-1 peak to its statistically evaluated AUC value. Abbreviations: Pro-Hyp, prolyl-hydroxyproline; PGC-1α, peroxisome proliferator-activated receptor-gamma coactivator 1-alpha; Foxg1, forkhead box G1; UCP-1, uncoupling protein 1; C/EBPα, CCAAT/enhancer-binding protein alpha; PPARγ, peroxisome proliferator-activated receptor gamma; GLP-1, glucagon-like peptide-1; RCT, randomized controlled trial; SEM, standard error of the mean; AUC, area under the curve; pmol/L, picomole per litre; pg/mL, picogram per millilitre.

**Figure 5:**
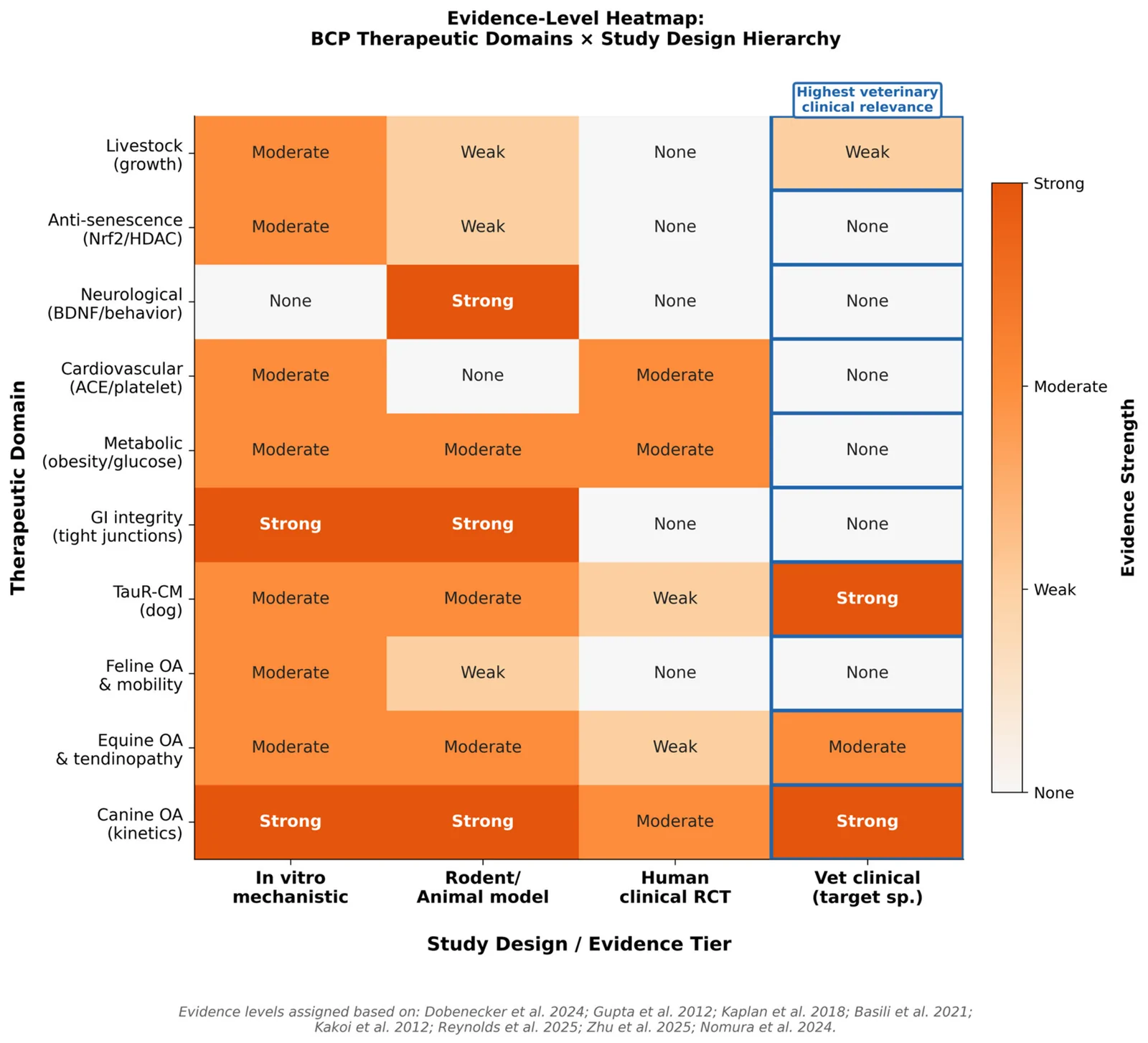
Evidence-level heatmap. Color-coded matrix displaying evidence strength (None → Weak → Moderate → Strong) across ten therapeutic domains and four study design tiers. Blue-bordered column highlights veterinary clinical evidence tier. See Section 7.1 for evidence tier definitions (Dobenecker et al., 2024; Gupta et al., 2012; Kaplan et al., 2018; Basili et al., 2021; Kakoi et al., 2012; Reynolds et al., 2025; Zhu et al., 2025; Nomura et al., 2024) [2,3,4,5,6,9,26,29]. Abbreviations: BCP, bioactive collagen peptide; OA, osteoarthritis; TauR-CM, taurine-responsive dilated cardiomyopathy; GI, gastrointestinal; ACE, angiotensin-converting enzyme; BDNF, brain-derived neurotrophic factor; Nrf2, nuclear factor erythroid 2-related factor 2; HDAC, histone deacetylase; RCT, randomized controlled trial; sp., species.

Table 3 provides a consolidated overview of the primary therapeutic domains addressed in this review, integrating target mechanisms, evidence stratification, and key references. The novel metabolic mechanisms underpinning these applications—incretin (GLP-1/PYY) modulation and Pro-Hyp-driven brown adipogenesis—are summarized in Figure 4, while the evidence-level heatmap across therapeutic domains and study design tiers is illustrated in Figure 5.

### 7.2. Translational Limitations and Methodological Considerations

Despite the breadth and mechanistic depth of the evidence summarized above, several translational limitations must be acknowledged to ensure scientifically responsible application of these findings in clinical veterinary practice.

First, the overwhelming majority of mechanistic data derives from in vitro cell culture systems or rodent models. While the high conservation of collagen receptors (integrins, DDR2, PEPT1/PEPT2) across mammalian species provides a biologically plausible basis for translational extrapolation, direct pharmacokinetic studies characterizing BCP tissue distribution in dogs, cats, and horses following oral administration remain limited [1].

Second, the dose–response relationships for BCP supplementation have not been systematically characterized across veterinary species. The 240 mg/kg body weight protocol employed in the Dobenecker 2024 canine OA trial [4] represents a single validated dosing regimen; whether lower doses achieve equivalent efficacy, or whether dose escalation produces proportional benefit, remains unknown.

Third, the microbiome-mediated mechanisms of BCP bioactivity—bile acid modulation, FXR/TGR5 signaling, GLP-1 secretion—depend on baseline microbiome composition, which varies substantially between individuals, breeds, and dietary backgrounds. Standardization of microbiome-outcome endpoints in future veterinary RCT design is a methodological priority. A subset of in vitro studies employing high concentrations of CH have also reported unexpected catabolic effects in cartilage explant models, attributable in part to endotoxin contamination of collagen research-grade preparations and to dose ranges that exceed physiological exposure [67].

Fourth, the literature is skewed toward positive results, and most of the studies on which this review rests carry a commercial tie. The canine and equine osteoarthritis trials were funded or supplied by a gelatin manufacturer whose product was the test article, with a company employee among the authors and a co-inventor on collagen peptide patents; both undenatured type II collagen trials in dogs were co-authored by the supplement manufacturer; and the brown adipogenesis and microbiome studies each carry manufacturer co-authorship. Trial registration is inconsistent, and null findings are seldom published. No formal assessment of publication bias in this field has been reported, so its magnitude cannot be estimated. None of this invalidates the individual findings, but the effect sizes summarized in the table above should be read as upper bounds.

### 7.3. Future Research Priorities

In the musculoskeletal domain, prospective randomized controlled trials using objective force-plate kinetics are needed in feline OA, where behavioral pain masking renders subjective endpoints unreliable. The combined UC-II plus BCP protocol, theoretically additive through orthogonal mechanisms, has not been tested in a rigorous comparative design.

In the cardiovascular domain, the relationship between dietary collagen amino acid content, plasma taurine status, and DCM risk in non-golden-retriever breeds requires systematic longitudinal investigation. Bile acid profiling—using the validated LC-MS/MS methodology [21]—should be incorporated as a secondary outcome in future TauR-CM trials, given that BA conjugation status reflects functional taurine availability in the enterohepatic compartment.

In the metabolic domain, the Pro-Hyp/PGC-1α/Foxg1 brown adipocyte differentiation pathway identified by Nomura et al. (2024) [29] requires in vivo confirmation in obese companion animal models. Finally, the neurological applications of low-molecular-weight BCPs in companion animals are essentially unexplored in clinical trials; the murine neurogenesis and anxiolytic data of Kakoi et al. (2012) [6] represent a compelling rationale for controlled behavioral trials in canine anxiety disorders and cognitive dysfunction syndrome.

Across species, comparability would be served by a minimum outcome set agreed in advance. In dogs and horses this would comprise objective kinetics—force-plate peak vertical force and vertical impulse—together with a validated owner- or handler-assessed instrument; in cats, accelerometry, because behavioural pain masking makes subjective endpoints unreliable. A standardized serum cartilage biomarker panel should be reported alongside the clinical endpoints. Where the BA hypothesis is under test, paired faecal microbiome and serum or faecal BA profiling by validated LC-MS/MS should be collected at the same time points. Every trial should report the full characterization of the collagen preparation used: source species, hydrolysis conditions, peptide molecular-weight distribution, quantified Pro-Hyp and Hyp-Gly content, and endotoxin concentration. Without this, a null result cannot be distinguished from an inactive batch.

## 8. Conclusions

The literature reviewed here suggests that the bioactivity of orally administered bioactive collagen peptides is not confined to the musculoskeletal compartment. Effects reported in the gastrointestinal, endocrine, cardiovascular, neurological, and epigenetic domains have been proposed to converge on the enterohepatic bile acid pool as a shared node. That convergence is a working model assembled from separate experimental systems rather than an established pathway, and it remains to be tested directly. Collagen peptides are proposed to reach this pool through microbiome-driven secondary bile acid remodeling and FXR/TGR5 signaling; the parallel rise in GLP-1 and PYY after collagen intake is not yet shown to depend on this bile acid route. Taurine reaches the same pool through conjugation capacity. These are parallel dietary inputs to one compartment rather than a single collagen-controlled pathway.

In the veterinary clinical domain, the highest level of evidence currently supports the use of specific BCPs in canine and equine osteoarthritis, with objective force-plate kinetic validation, and dietary taurine intervention in breed-specific dilated cardiomyopathy, with echocardiographic outcome data. In these two domains the intervention does more than relieve symptoms: canine and equine osteoarthritis show measurable kinetic improvement, and taurine-responsive dilated cardiomyopathy can reverse echocardiographically with taurine repletion. The osteoarthritis effect is symptomatic and acts at the matrix level rather than being curative, and the cardiomyopathy reversal is attributable to taurine, not to collagen peptides.

Collagen peptides and taurine are not interchangeable nutritional strategies. A high-collagen or gelatin-rich diet supplies abundant glycine, proline, and hydroxyproline but no taurine precursors, and it cannot raise taurine status or treat taurine-responsive cardiomyopathy. Taurine supplementation, conversely, supplies none of the amino acids that underlie the musculoskeletal effects of collagen hydrolysate. The two converge on one compartment and are prescribed for separate indications; substituting one for the other has no rational basis.

Recent mechanistic data on brown adipocyte thermogenesis, hippocampal neurogenesis, platelet function, and anti-senescent epigenetic reprogramming extend the plausible scope of BCP effects, but these remain preclinical and require veterinary in vivo confirmation before clinical claims are warranted.

## Figures and Tables

**Table 1:** Mechanism-level evidence map for the gut–collagen peptide axis and related pathways. Highest direct evidence refers to the strongest study design in which the mechanism itself—not a downstream clinical outcome—was measured. Shown after oral CH? asks whether the mechanism was demonstrated following oral administration of collagen hydrolysate, as distinct from direct exposure of cells or tissues. Within the table, upward arrows (↑) indicate an increase or elevation in microbial abundance or metabolic synthesis, whereas rightward arrows (→) denote a sequential mechanistic cascade or directional pathway from molecular trigger to physiological outcome. Abbreviations: CH, collagen hydrolysate; UC-II, undenatured type II collagen; BSH, bile salt hydrolase; BA, bile acid; FXR, farnesoid X receptor; TGR5, Takeda G-protein-coupled receptor 5; GLP-1, glucagon-like peptide-1; PYY, peptide YY; ACE, angiotensin-converting enzyme; OA, osteoarthritis; RCT, randomized controlled trial.

Proposed Mechanism	Highest Direct Evidence	Species Demonstrated	Shown After Oral CH?	Status
Tight-junction protein rescue (ZO-1, occludin)	In vitro + rodent colitis model	Human cell line, mouse, rat	Yes (rodent, dietary)	Demonstrated
MLCK-dependent suppression of cytokine-driven barrier loss	In vitro	Human cell line	No	Demonstrated (direct exposure)
Proposed mechanism	Highest direct evidence	Species demonstrated	Shown after oral CH?	Status
↑ BSH-carrying taxa	None (inferred)	—	No	Hypothetical
↑ Secondary BA synthesis	None (inferred)	—	No	Hypothetical
FXR/TGR5 activation by collagen-derived BA change	None (inferred)	—	No	Hypothetical
GLP-1/PYY release after collagen or gelatin load	Human RCT	Human	Yes	Demonstrated; mechanism unresolved
Pro-Hyp → Foxg1 → PGC-1α → brown adipogenesis	In vitro (C3H10T1/2)	Mouse cell line	No	Demonstrated (direct exposure)
α2β1/DDR2-driven chondrocyte matrix anabolism	In vitro, triple-helical ligand	Human, bovine, murine cells	No	Demonstrated (direct exposure)
UC-II oral tolerance via GALT and Treg induction	Veterinary RCT (clinical outcome)	Dog, horse	Not applicable (UC-II)	Demonstrated (outcome); mechanism inferred
Taurine → BA conjugation → enterohepatic homeostasis	Feline depletion model; canine clinical series	Cat, dog	Not applicable (taurine)	Demonstrated
ACE inhibition by marine collagen peptides	In vitro enzyme assay	Cell-free, human cell line	No	Demonstrated (direct exposure); no in vivo data
Keap1–Nrf2/HDAC modulation	In vitro, purified or fractionated peptides	Human cell lines	No	Demonstrated (direct exposure)

**Table 2:** Species-specific plasma and whole blood taurine reference ranges and dilated cardiomyopathy (DCM) risk classification in veterinary-relevant species. Values represent published reference intervals; deficiency thresholds are based on consensus criteria from cardiology and nutrition literature. Whole blood and plasma taurine are expressed in nmol/mL (1 nmol/mL = 1 μmol/L). Abbreviations: DCM, dilated cardiomyopathy; TauR-CM, taurine-responsive dilated cardiomyopathy; WB, whole blood; plasma, EDTA plasma; RI, reference interval; n/a, not applicable or not established; n.d., not determined.

Species/Breed	WB Taurine (nmol/mL) Reference	Plasma Taurine (nmol/mL) Reference	Deficiency Threshold	TauR-CM Risk	Primary Dietary Risk Factor	Key Reference
Dog (general)	200–350	60–120	WB < 200; plasma < 40	Breed-dependent	Grain-free/legume-rich diet	Kaplan et al., 2018 [2]
Golden Retriever	Often < 200 (affected)	Often < 40 (affected)	WB ≤ 250; plasma ≤ 60	High	Low methionine/cysteine, grain-free	Kaplan et al., 2018 [2]
English Cocker Spaniel	n/a	Reduced vs. control	Plasma < 50 (RI 50–180)	High	not characterized (diet history unavailable)	Basili et al., 2021 [3]
Doberman Pinscher	Not established	Not established	n.d.	Very low; DCM is genetic and taurine-independent (PDK4 variant)	None established (genetic)	Meurs et al., 2012 [47]
Boxer	Not established	Not established	n.d.	Very low; DCM associated with the striatin deletion	None established (genetic)	Meurs et al., 2013 [48]
Cat (obligate carnivore)	300–600	60–120	WB < 200; plasma < 40	Very High (if taurine-deficient diet)	Taurine-free or cereal-based diet	[49]
Horse	n/a	n/a	n.d.	Not established	n/a	—
Human	164–318	44 ± 9 (fasting)	n/a (not standardized)	Not applicable	Low dietary taurine, diabetes	[50]

Breed-specific taurine reference intervals have not been established for the Doberman Pinscher or the Boxer; the entries reflect the absence of systematically reported values rather than normal findings. In the Doberman Pinscher, familial DCM is associated with a PDK4 splice-site variant and, in dogs lacking it, with a titin variant; genetic heterogeneity remains. In the Boxer, 30 of 33 dogs with DCM carried the striatin 3′-UTR deletion, and 3 did not, indicating at least one further cause. Neither breed presentation is taurine-responsive.

**Table 3:** Summary of therapeutic applications for bioactive collagen peptides and taurine-related interventions. Abbreviations: MMP, matrix metalloproteinase; PVF, peak vertical force; CBPI, Canine Brief Pain Inventory; FXR, farnesoid X receptor; TGR5, Takeda G-protein-coupled receptor 5; GLP-1, glucagon-like peptide-1; PYY, peptide YY; ACE, angiotensin-converting enzyme; NO, nitric oxide; ET-1, endothelin-1; IDDM, insulin-dependent diabetes mellitus; TauR-CM, taurine-responsive dilated cardiomyopathy; CHF, congestive heart failure; AChE, acetylcholinesterase; BDNF, brain-derived neurotrophic factor; SA-β-Gal, senescence-associated beta-galactosidase; MSC-EV, mesenchymal stem cell-derived extracellular vesicle. **Symbols:** →, downstream signaling pathway or activation cascade; ↑, upregulation or increased concentration; ↓, downregulation or reduction.

Therapeutic Domain	Primary Target Mechanism	Evidence Base	Key References	Evidence Tier
Canine and Equine OA	α2β1 integrin → type II collagen/aggrecan synthesis; MMP suppression	Vet clinical: significant improvement in PVF and vertical impulse; CBPI quality-of-life score improved vs. omega-3 com-parator; CBPI pain score unchanged	Dobenecker et al., 2018 [36]; Dobenecker et al., 2024 [4]; Gupta et al., 2012 [5]	Strong (canine); Moderate (equine)
Metabolic Syndrome and Obesity	Gut microbiota → FXR/TGR5 activation; GLP-1/PYY upregulation; Pro-Hyp → PGC-1α/Foxg1 brown adipogenesis	Animal Model/Human: Reduction in visceral fat; improved glucose tolerance; appetite suppression	Reynolds et al., 2025 [26]; López-Yoldi et al., 2025 [15]; Nomura et al., 2024 [29]	Moderate (in vitro/animal); None (vet clinical)
Cardiovascular Disease	ACE inhibition (GPPGPPGL); platelet taurine restoration; NO ↑, ET-1 ↓	In vitro/Human: Decreased hypertension parameters; normalized platelet aggregation in IDDM	Xiang et al., 2024 [54]; Franconi et al., 1995 [51]; Iwaniak et al., 2014 [53]	Moderate
Gastrointestinal Integrity	TNF-α suppression; MLCK modulation; ZO-1/occludin rescue	In vitro/Animal Model: Epithelial barrier restoration in stress-induced colitis	Chen et al., 2017 [8]; Zhu et al., 2025 [9]	Strong (in vitro/animal)
TauR-CM (dog)	Taurine → bile acid conjugation; mitochondrial membrane stabilization; FXR/TGR5 restoration	Vet Clinical: Echocardiographic improvement and CHF reversal in golden retrievers and cocker spaniels	Kaplan et al., 2018 [2]; Basili et al., 2021 [3]; Miyazaki et al., 2020 [18]	Strong
Neurological Repair and Behavior	ROS scavenging; AChE inhibition; BDNF/p-CREB upregulation; hippocampal neurogenesis	Animal Model: Rescue of cognitive/motor functions post-asphyxia; reduced anxiety in mice	Xu et al., 2015 [62]; Kakoi et al., 2012 [6]	Strong (animal); None (vet clinical)
Dermal Anti-Aging and Wound Healing	HDAC/HAT modulation; Keap1–Nrf2/HO-1 axis; synergy with MSC-EVs	In vitro/Human: SA-β-Gal marker reduction; cortisol-induced fibroblast damage reversal	Zhu et al., 2024 [65]; Chae et al., 2021 [63]; Lin et al., 2023 [64]	Moderate
Livestock Production	Rate-limiting AA provision (Gly/Pro); integrin/DDR2 receptor signaling	Translational: Evidence from companion animal and rodent models; direct livestock RCT data absent	No direct livestock RCT evidence available (see Section 3.5)	Weak (vet clinical)

## Data Availability

No new data were created or analyzed in this study. Data sharing is not applicable to this article.

## Abbreviations

The following abbreviations are used in this manuscript:

ACE	angiotensin-converting enzyme
AChE	acetylcholinesterase
AUC	area under the curve
BA	bile acid
BCP	bioactive collagen peptide
BDNF	brain-derived neurotrophic factor
BSH	bile salt hydrolase
C/EBPα	CCAAT/enhancer-binding protein alpha
CAF	cancer-associated fibroblast
CAT	catalase
CBPI	Canine Brief Pain Inventory
CH	collagen hydrolysate
CHF	congestive heart failure
DCA	deoxycholic acid
DCM	dilated cardiomyopathy
ECM	extracellular matrix
ET-1	endothelin-1
Foxg1	forkhead box G1
FS	fractional shortening
FXR	farnesoid X receptor
GALT	gut-associated lymphoid tissue
GLP-1	glucagon-like peptide-1
GPBAR1	G-protein-coupled bile acid receptor 1
GSH	glutathione
GSSG	oxidized glutathione
HAT	histone acetyltransferase
HDAC	histone deacetylase
HIF1α	hypoxia-inducible factor 1-alpha
HO-1	heme oxygenase-1
HSC	hepatic stellate cell
Hyp	hydroxyproline
Hyp-Gly	hydroxyprolyl-glycine
IBD	inflammatory bowel disease
IC50	half-maximal inhibitory concentration
IDDM	insulin-dependent diabetes mellitus
IRS-1	insulin receptor substrate-1
ITIM	immunoreceptor tyrosine-based inhibitory motif
kDa	kilodalton
Keap1	Kelch-like ECH-associated protein 1
LAIR-1/2	leukocyte-associated immunoglobulin-like receptor 1/2
LCA	lithocholic acid
LC-MS/MS	liquid chromatography-tandem mass spectrometry
LMW	low molecular weight
LPS	lipopolysaccharide
LVIDd	left ventricular internal diameter in diastole
MAPK	mitogen-activated protein kinase
MLCK	myosin light chain kinase
MMP	matrix metalloproteinase
MSC-EV	mesenchymal stem cell-derived extracellular vesicle
NAFLD	non-alcoholic fatty liver disease
NASH	non-alcoholic steatohepatitis
NF-κB	nuclear factor kappa-light-chain-enhancer of activated B cells
NLRP3	NLR family pyrin domain-containing protein 3
NO	nitric oxide
Nrf2	nuclear factor erythroid 2-related factor 2
OA	osteoarthritis
P1NP	amino-terminal propeptide of type I collagen
PEPT1/2	peptide transporter 1/2 (SLC15A1/A2)
PGC-1α	peroxisome proliferator-activated receptor gamma coactivator 1-alpha
PPARγ	peroxisome proliferator-activated receptor gamma
Pro	proline
Pro-Hyp	prolyl-hydroxyproline
PVF	peak vertical force
PYY	peptide YY
ROS	reactive oxygen species
SASP	senescence-associated secretory phenotype
SA-β-Gal	senescence-associated beta-galactosidase
SHP-1/2	Src homology 2-containing protein tyrosine phosphatase 1/2
SOD	superoxide dismutase
TauR-CM	taurine-responsive dilated cardiomyopathy
TGF-β	transforming growth factor-beta
TGR5	Takeda G-protein-coupled receptor 5
TIMP	tissue inhibitor of metalloproteinases
TNF-α	tumor necrosis factor-alpha
Treg	regulatory T cell
UC-II	undenatured type II collagen
UCP-1	uncoupling protein 1
UDCA	ursodeoxycholic acid
WB	whole blood
ZO-1	zonula occludens-1
